# VHL胚系突变所致家族性红细胞增多症2型1例报告并文献复习

**DOI:** 10.3760/cma.j.cn121090-20241011-00390

**Published:** 2025-01

**Authors:** 宁宁 柳, 丽娟 潘, 志坚 肖, 泽锋 徐

**Affiliations:** 1 中国医学科学院血液病医院（中国医学科学院血液学研究所），血液与健康全国重点实验室，国家血液系统疾病临床医学研究中心，细胞生态海河实验室，天津 300020 State Key Laboratory of Experimental Hematology, National Clinical Research Center for Blood Diseases, Haihe Laboratory of Cell Ecosystem, Institute of Hematology & Blood Diseases Hospital, Chinese Academy of Medical Sciences & Peking Union Medical College, Tianjin 300020, China; 2 天津医学健康研究院，天津 301600 Tianjin Institutes of Health Science, Tianjin 301600, China

**Keywords:** 遗传性红细胞增多症, 家族性红细胞增多症2型, VHL基因, 胚系突变, Hereditary erythrocytosis, Familial erythrocytosis type 2, VHL gene, Germline mutation

## Abstract

**目的:**

加深对VHL基因复合杂合突变导致的家族性红细胞增多症2型（ECYT2）疾病的认识。

**方法:**

回顾性分析1例ECYT2患者的病例资料，探讨ECYT2的发病机制、临床特点、诊治及预后，并对相关文献进行复习。

**结果:**

男，31岁，因“颜面部和双手潮红29年”入院，全外显子组测序检出VHL p.P81L和p.N90T复合杂合突变，其父母均只携带其中1个杂合突变，为正常表型。结合患者血液学相关化验检查，明确诊断ECYT2，予以红细胞单采术和阿司匹林100 mg/d治疗后，患者因血液淤滞造成的头晕、头痛症状缓解，期间无血栓及出血事件发生。

**结论:**

ECYT2是一类少见的常染色体隐形遗传性疾病，本例VHL基因复合杂合突变所致ECYT2为国内首次报道。其临床特点为红细胞容量升高，血清促红细胞生成素水平正常或升高以及血红蛋白氧亲和力水平正常，易引起血栓和出血性并发症，导致早期死亡。

## Abstract (en)

**Objective:**

To enhance the understanding of familial erythrocytosis type 2（ECYT2）resulting from compound heterozygous mutations in the VHL gene.

**Methods:**

We conducted a retrospective analysis of the case data from a patient with ECYT2 to investigate its pathogenesis, clinical features, diagnosis and treatment options, as well as prognosis, while also reviewing the relevant literature.

**Results:**

A 31-year-old man was admitted to the hospital due to facial and hand flushing that had persisted for 29 years. Whole exome sequencing revealed compound heterozygous mutations in VHL p.P81L and p.N90T. Both of his parents were found to carry only one of these heterozygous mutations, yet they exhibited normal phenotypes. Based on the patient's hematological tests, a clear diagnosis of ECYT2 was established. Following treatment with erythrocytapheresis and daily administration of aspirin at a dosage of 100 mg, the patient experienced relief from dizziness and headaches associated with blood hyperviscosity, without any thrombotic or bleeding complications during this period.

**Conclusion:**

ECYT2 is a rare group of autosomal recessive genetic disorders. This case of ECYT2, resulting from compound heterozygous mutations in the VHL gene, represents the first report in China. Clinically, it is characterized by elevated red cell mass, normal or increased serum erythropoietin levels, and normal hemoglobin oxygen affinity levels. These factors contribute to thrombotic and bleeding complications that can lead to early mortality.

红细胞增多症可分为原发性和继发性，继发性红细胞增多症多由促红细胞生成素（EPO）增多引起，可进一步分为遗传性和获得性[1]。特发性红细胞增多症是除外已知的原发性或继发性红细胞增多症的异质性疾病，发病机制尚不明确[1]–[2]。HIF-PHD-VHL信号转导通路异常所致的红细胞增多症为遗传性红细胞增多症。其中，家族性红细胞增多症2型（ECYT2）是VHL双等位基因胚系突变所致的常染色体隐性遗传性疾病［在线人类孟德尔遗传（OMIM）IDs: 263400］，以VHL c.598C>T（p.R200W）纯合突变的Chuvash红细胞增多症（CP）最为典型[3]。国外VHL基因复合杂合突变所致ECYT2少见，国内尚未见报道。我们新近发现1例，现报道如下并对相关文献进行复习。

## 病例资料

患者，男，31岁，主因“颜面部和双手潮红29年”于2022年7月入我院。患者1993年7月无明显诱因出现颜面部和双手潮红，偶有头晕、头痛，在当地医院查血常规示RBC增多，WBC和PLT正常（具体不详），腹部超声提示脾脏肿大（具体不详），当地考虑为“红细胞增多症”，先后予阿司匹林、高三尖杉酯碱、干扰素、静脉放血、红细胞单采术等治疗，头晕、头痛等不适症状在静脉放血或红细胞单采术后可缓解，血常规波动情况：WBC（5.00～6.00）×10^9^/L，HGB 180～250 g/L，红细胞压积（Hct）55.0％～75.0％，PLT（100～200）×10^9^/L。2021年10月，患者再次就诊于当地医院，查血常规：WBC 5.00×10^9^/L，RBC 9.09×10^12^/L，HGB 194 g/L，Hct 67.6％，PLT 200×10^9^/L。EPO：618 U/L（正常参考值2.59～18.50 U/L）。髂骨穿刺骨髓涂片分类：增生活跃，粒系比例0.390，红系比例0.355，淋巴细胞比例0.220，全片见巨核细胞63个。骨髓组织病理学检查示：骨髓增生大致正常（60％），粒红比例略减小，粒红系细胞均以偏成熟阶段为主，巨核细胞数量及形态大致正常，以分叶核为主；少量淋巴细胞。网状纤维染色MF-0级。BCR∷ABL融合基因阴性。应用二代测序（NGS）对髓系肿瘤基因突变（包括：JAK2 V617F、JAK2 exon12、CALR exon9和MPL exon10）进行筛查：均阴性。腹部超声：脾脏肿大（长13.3 cm，厚4.9 cm）。肾上腺CT未见异常。未明确诊断。予羟基脲1.0 g/d和阿司匹林100 mg/d口服治疗，入我院前的近半年，监测血常规波动情况：WBC（5.00～6.00）×10^9^/L，HGB 173～202 g/L，Hct 67.0％～69.0％，PLT（100～200）×10^9^/L。为明确诊断来我院就诊。

患者既往有“甲状腺功能亢进病史”5年，规律口服甲巯咪唑2.5 mg/d，无血栓及出血病史。患者两系三代中无近亲结婚；祖父及舅舅有腔隙性脑梗死病史，血常规无异常；无类似家族病史。无吸烟、饮酒史。入院查体：生命体征平稳，体格发育正常，双眼矫正视力0.5，颜面部及双手潮红，肝肋缘下未触及，脾下缘在左锁骨中线与左肋缘交点下3 cm，余未见明显异常。入院时血常规：WBC 5.34×10^9^/L，RBC 7.21×10^12^/L，HGB 178 g/L，Hct 59.2％，PLT 214×10^9^/L。血生化检查：肝肾功能大致正常，乳酸脱氢酶235.1 U/L（正常参考值0～247 U/L），尿酸573.9 µmol/L（正常参考值208.3～428.4 µmol/L）。凝血功能大致正常。血清铁5.86 µmol/L（正常参考值8.9～32.3 µmol/L），转铁蛋白饱和度0.09（正常参考值0.25～0.50），总铁结合力和未饱和铁正常。铁蛋白6.6 µg/L（正常参考值23.9～336.2 µg/L），EPO 1 100.96 U/L（正常参考值2.59～18.50 U/L）。EPO依赖性红系爆式集落形成单位（BFU-E）20/10^5^ BMMNC［正常参考值（25～37）/10^5^ BMMNC］，非EPO依赖性BFU-E 0/10^5^ BMMNC（正常参考值0/10^5^ BMMNC）。细胞因子（流式检查）：IL-6 5.86 ng/L（正常参考值≤5.3 ng/L），TNF-α 4.81 ng/L（正常参考值≤4.6 ng/L）。髂骨穿刺骨髓涂片分类：增生活跃，粒系比例0.335，红系比例0.460，成熟淋巴细胞比例0.195，单核细胞比例0.010，全片共见巨核细胞29个。免疫组织化学染色（CD41）：全片巨核细胞19个，正常巨核细胞18个，双核巨核细胞1个。骨髓组织病理学检查：增生大致正常（40％～50％），粒红比例略减小，粒系各阶段细胞可见，红系以中晚幼红细胞为主，巨核细胞散在分布，分叶核为主，网状纤维染色MF-1级。染色体核型：46,XY[20]。全外显子组测序（血液系统疾病方向），外周静脉血标本检测到VHL exon1 c.242C>T（p.P81L）和exon1 c.269A>C（p.N90T）复合杂合突变。对先证者及其父母进行家系验证，确认c.242C>T来自其父，c.269A>C来自其母，其父母均只携带其中1个杂合突变，为正常表型。参考美国医学遗传学与基因组学会（ACMG）和分子病理学协会（AMP）发布的遗传变异解读及报告指南[4]，上述突变均为可疑致病性/病理性突变。患者肿瘤标志物、ENA抗体谱、血红蛋白组分分析等其他化验检查无异常发现。腹部超声：胆囊多发结石，脾脏中度肿大（长15.3 cm，厚5.4 cm，肋缘下4.3 cm×3.0 cm）。泌尿系超声：双肾未见明显异常。超声心动图：二尖瓣前叶瓣尖略增厚（建议随诊），三尖瓣少量反流（流速正常范围内），无肺动脉高压。诊断：ECYT2。予红细胞单采术去除体内过多的红细胞，口服阿司匹林100 mg/d预防血栓。出院后继续口服阿司匹林100 mg/d，出现头晕、头痛等不适时行红细胞单采，末次红细胞单采时间为2022年7月13日。截至2024年6月末次随访时，患者无血栓及出血事件发生，血常规波动情况：WBC（5.00～7.00）×10^9^/L，HGB 150～170 g/L，Hct 50.0％～65.0％，PLT（200～300）×10^9^/L。

## 讨论及文献复习

ECYT相较于真性红细胞增多症（PV）而言是一类少见的遗传性疾病，OMIM（https://www.omim.org/）数据库根据其突变的基因不同将其分为8种亚型，ECYT1（OMIM IDs: 133100）是促红细胞生成素受体（EPOR）基因杂合突变所致。ECYT 2～5（OMIM IDs: 263400、609820、611783和617907）分别是VHL、EGLN1、EPAS1和EPO基因突变所致。ECYT 6～8（OMIM IDs: 617980、617981和222800）的病因是血红蛋白β（HBB）、血红蛋白α-1/2（HBA1/HBA2）和BPGM基因突变导致血红蛋白对氧气的亲和力增加。除VHL和BPGM基因突变相关的ECYT2和ECYT8是常染色体隐性遗传外，其余ECYT都是常染色体显性遗传。美国梅奥诊所从446例JAK2（exon12～15）突变阴性的红细胞增多症患者中筛查出7例（1.6％）伴VHL胚系突变[5]。Chandrasekhar等[6]对来自印度南部的65例JAK2（exon12和14）突变阴性的红细胞增多症患者进行筛查，伴VHL胚系突变共有6例（9.2％）。Lee等[7]韩国学者从21例JAK2（exon12～14）突变阴性的红细胞增多症患者中筛查出1例（4.8％）伴VHL胚系突变。而我国对于ECYT2仅有1例病例报道[8]。

人类VHL基因位于染色体3p25-26，包含3个外显子（E1、E2和E3）和1个隐性外显子（E1′），编码4种不同的蛋白异构体（pVHL-213、pVHL-160、pVHL-172和pVHL-X1）[9]。EPO基因的表达受HIF的调节，HIF是一种异源二聚体蛋白，由HIF-1α、HIF-2α、HIF-3α亚基和HIF-1β亚基组成，在氧气正常条件下，PHD通过将氧原子插入脯氨酸残基（人类HIF-1α的Pro 402或Pro 564）中来修饰HIF-α亚基，羟化后的HIF-α亚基和VHL蛋白结合，后者靶向HIF-α亚基泛素化和蛋白酶体降解，从而减少EPO的产生[10]，而在缺氧条件下，PHD活性降低使HIF-α羟化减少，阻碍其与VHL蛋白结合，HIF积聚导致包括EPO在内的多个下游靶基因表达增强[3]。此外，VHL p.R200W突变导致VHL蛋白构象发生改变，增强与SOCS1的结合，从而抑制JAK2结合和降解，导致JAK2/STAT5信号通路过度激活，这也是CP患者的红系祖细胞对EPO超敏感的原因[11]。

VHL基因作为一种抑癌基因，通过参与HIF的降解抑制肿瘤的生长、侵袭和转移，VHL基因突变导致ECYT2和多种血管性肿瘤（如VHL综合征，OMIM IDs：193300）的发生[12]。当存在VHL基因突变时，应仔细鉴别ECYT2和VHL综合征。ECYT2是VHL双等位基因胚系突变引起HIF-PHD-VHL信号转导通路异常所致，为常染色体隐性遗传性疾病，其中CP最具有代表性。1974年有学者首次描述了Chuvash出现的家族性红细胞增多症，随后于2002年发现3号染色体短臂2区上的VHL基因错义突变导致第200位的精氨酸被色氨酸取代（p.R200W）是CP的分子遗传学基础[3],[13]。VHL综合征为常染色体显性遗传性疾病，其亦是由VHL基因突变所致，常表现为各种良性和恶性肿瘤（如视网膜毛细血管瘤、小脑和脊髓血管母细胞瘤、肾细胞癌、嗜铬细胞瘤和胰腺肿瘤），与Knudson提出的“二次打击”学说有关，即VHL基因的首次突变发生于生殖细胞，使个体具有遗传易感性；第二次突变发生于体细胞，促进肿瘤的发生发展[14]。而几乎所有的ECYT2患者都存在VHL双等位基因胚系突变，这可能是同为VHL基因突变为何会出现两种截然不同表型的原因。除此之外，二者在临床表现上也大不相同。VHL综合征中红细胞增多少见，与具有分泌功能的肿瘤产生EPO有关[15]–[16]；ECYT2患者罹患VHL综合征相关肿瘤的概率极低，仅见于少数病例报道[17]–[18]。ECYT2通常无眼部病变，而VHL综合征的眼部病变多为视网膜毛细血管瘤，极少数也可为视神经成血管细胞瘤，病变多为单侧，患者出现视力下降，眼底检查可见视神经萎缩[19]–[20]。本例患者自幼视物模糊，双眼矫正视力为0.5，无视物变形，眼科完善检查提示视神经发育不良。与上述VHL综合征眼部病变不符，视力问题首先应考虑为先天性眼底发育异常。

ECYT2的特征是红细胞容量（Red cell mass, RCM）升高，血清EPO水平正常或升高以及血红蛋白氧亲和力水平正常，易引起血栓和出血性并发症，导致早期死亡[21]。其具有与原发性红细胞增多症相一致的红系造血祖细胞对EPO超敏感的特点[3]，同时也具有类似于继发性红细胞增多症的EPO水平正常或升高的特点[22]。部分患者可出现脾脏肿大[23]–[25]，既往对VHL^R200W/R200W^突变小鼠研究发现VHL p.R200W突变促进了红系分化，且脾脏而非骨髓是此类小鼠红细胞生成增多的主要来源，这可能是部分患者会出现脾脏肿大的原因[26]。虽然CP患者红系祖细胞对EPO超敏感，但亦有报道p.H191D和p.D126N等纯合突变的ECYT2患者红系祖细胞对EPO不敏感[27]–[28]。

ECYT2最常见的胚系突变是VHL p.R200W，Liu等[29]为了探究此突变是否发生在同一祖先或是由于重复发生的突变事件，对来自不同国家共101例VHL p.R200W突变者和447例健康对照者的样本进行了单倍型分析，得出VHL p.R200W突变起源于14 000～62 000年前的同一祖先。但随后Cario等[30]发现VHL p.R200W突变并非仅由同一祖先传播，土耳其一个红细胞增多症家系的VHL p.R200W突变为独立发生。部分ECYT2为VHL p.R200W与其他VHL突变形成的复合杂合子（如p.V130L、p.G144R、p.L188V和p.P192A）[22],[31]–[32]，也可为除VHL p.R200W之外的纯合子（如p.D126N、p.P138L和p.H191D）[22],[28],[33]或复合杂合子（如p.R79C/p.L188V和p.T124A/p.L188V）[34]–[35]。曾有学者报道少数ECYT2仅有一个VHL等位基因突变，如在一个乌克兰家系中，检出父亲和有红细胞增多的姐弟二人均为VHL p.D126Y等位基因胚系突变，其父亲没有红细胞增多，随后也排除了该姐弟2人从其母亲处遗传无效VHL等位基因的可能，其导致红细胞增多表型的分子机制有待进一步研究[31]。本例患者存在VHL p.P81L和p.N90T胚系突变，为少见的非p.R200W复合杂合突变。p.P81L在既往的VHL综合征相关文献中报道过，表现为肾细胞癌和嗜铬细胞瘤，其中的病例均无红细胞增多表现[36]–[37]；而p.N90T既往未见相关文献报道。

目前尚无特殊手段治疗ECYT2，在一项CP组（155例）和对照组（154例）患者年龄、性别、居住地匹配的前瞻性研究[38]中，CP患者新发血栓事件率［23.9％（37/155）］高于对照组［3.2％（5/154）］；多因素分析中，年龄更大、发生过血栓事件和接受过静脉放血治疗是CP患者更容易新发血栓事件的独立预测因素；且在新发血栓事件的CP患者中，近半数患者［45.5％（15/33）］入组后口服阿司匹林75 mg/d，与无新发血栓事件的CP患者中口服阿司匹林人数［36.1％（44/122）］相比，差异无统计学意义（*P*＝0.52），表明低剂量阿司匹林对CP患者并无显著保护作用。但对于非VHL p.R200W突变的ECYT2患者来说，目前尚无研究表明阿司匹林无法预防血栓。Gangat等[21]认为对于HIF-PHD-VHL信号转导通路异常所致的红细胞增多症患者同样应严格控制心血管风险和抗血小板治疗，故本例ECYT2患者我们给予常规口服阿司匹林预防血栓。另一项研究中，CP组患者的总死亡率［46.9％（45/96）］高于PV组患者［18.5％（225/1213）］（*P*<0.001）。尽管与PV组患者相比，CP组患者的中位年龄更小（16岁对60岁），但CP组患者中因脑血管事件或外周血栓事件而死亡的比例却更高［46.7％（21/45）对21.9％（42/192），*P*＝0.001］。需要注意的是，CP组患者随访时间皆超过23年，而90％的PV组患者随访时间短于20年[23]。该研究中78.2％（68/87）的CP组患者接受了静脉放血治疗，但相关回归分析表明其与降低血栓风险（*P*＝0.12）和死亡率（*P*＝0.2）无关，这表明血栓事件可能与Hct升高以外的因素有关[23]。既往对CP的小鼠模型进行研究发现肺动脉高压可能与HIF-2α的活性增加导致肺部血管重塑有关[26]，并且HIF-α的负性调控因子PHD的活性需要铁的作用[39]。有研究者对CP患者行超声心动图检查，发现与对照组（27例）相比，CP组患者（82例）的确具有更高的肺动脉压力［三尖瓣反流速度（2.50±0.03）m/s对（2.30±0.05）m/s，*P*＝0.005］，较低的铁蛋白水平与静脉放血有关，是更高的肺动脉压力的独立预测因素（*β*＝0.29，*P*＝0.009）[40]。尽管静脉放血治疗对CP患者无益甚至可能会引起血栓和使肺动脉压力升高，但它仍是缓解因Hct升高而出现头痛、肌肉痉挛等不适的主要措施[41]。细胞毒性药物亦可用于降低Hct，但其有白血病转化风险，故静脉放血治疗仍为优先考虑，且与PV的治疗不同的是，放血时不应追求Hct下降程度，应以患者症状减轻程度为准[21],[42]。如前所述，VHL突变会导致JAK2/STAT5通路的过度激活，有学者构建了VHL^R200W/R200W^突变小鼠，并对小鼠使用高选择性JAK2抑制剂TG101209，结果显示，与空白对照组相比，用TG101209治疗5周的小鼠Hct水平更低（35.4％对49.4％，*P*<0.05），小鼠的脾脏明显小于对照组，用药组小鼠脾脏中的巨核细胞数量也明显少于空白对照组（*P*<0.005）[11]。据此，Zhou等[43]尝试用JAK2抑制剂芦可替尼治疗3例CP患者，患者的血液学和头痛、腹痛等不适症状均有所改善，且无重大安全问题或严重不良反应，但对血栓事件和生存的影响目前尚不明确。上述结果表明，多数情况下ECYT2可不用治疗，只有当红细胞增多出现不适症状时，可谨慎尝试静脉放血或红细胞单采术治疗，且应以症状改善而非Hct下降为目标。此外，JAK2抑制剂芦可替尼可能对改善ECYT2患者的血液瘀滞所致症状和缩小脾脏有效，但尚需多中心、大样本量的临床研究来进一步验证其疗效和安全性。

本例伴VHL胚系突变的ECYT2青年患者，无红细胞增多的家族史，幼时起病，有脸红、手红、头晕和头痛等红细胞增多所致的临床表现，发病时为红细胞增多，Hct及HGB升高，血清EPO水平升高，红系祖细胞对EPO不敏感。患者存在VHL p.P81L和p.N90T复合杂合突变，其父母分别携带其中1个杂合突变且表型正常，为隐性遗传。ECYT2诊断明确。考虑到本例患者有血液淤滞所致的头晕和头痛等不适症状，予其行红细胞单采术缓解症状并口服阿司匹林100 mg/d预防血栓。该患者出院后密切监测血常规，长期口服阿司匹林，间断行红细胞单采术缓解不适症状，至今未出现血栓及出血事件。若该患者后期出现脾脏肿大进行性加重和体质性症状，可应用芦可替尼治疗。

综上所述，ECYT2是少见的常染色体隐性遗传性疾病，VHL p.R200W纯合突变所致CP占多数，具有地方性特点。我们报道了国内首例VHL基因p.P81L和p.N90T复合杂合突变导致ECYT2的病例，其中p.N90T既往未见报道。ECYT2患者只有在出现血液淤滞所致相关症状时，可谨慎考虑静脉放血或红细胞单采治疗。与PV患者相比，ECYT2患者无论是总死亡率还是血栓致死率皆更高，提示临床医师对ECYT2患者应密切监测，进行严格的心血管风险管理和抗血小板治疗，且对其发生血栓事件的相关因素需做进一步的探索。
